# Marginalized mixture models for count data from multiple source populations

**DOI:** 10.1186/s40488-017-0057-4

**Published:** 2017-04-07

**Authors:** Habtamu K. Benecha, Brian Neelon, Kimon Divaris, John S. Preisser

**Affiliations:** 1 National Agricultural Statistics Service, USDA, Washington, 20250 DC USA; 2 Department of Public Health Sciences, Medical University of South Carolina, Charleston, 29425 SC USA; 3 Departments of Epidemiology and Pediatric Dentistry, University of North Carolina, Chapel Hill, 27599-7450 NC USA; 4 Department of Biostatistics, University of North Carolina, Chapel Hill, 27599-7420 NC USA

**Keywords:** Dental caries, Excess zeros, Marginal inference, Mixture model, Over-dispersion, Zero-inflation

## Abstract

Mixture distributions provide flexibility in modeling data collected from populations having unexplained heterogeneity. While interpretations of regression parameters from traditional finite mixture models are specific to unobserved subpopulations or latent classes, investigators are often interested in making inferences about the marginal mean of a count variable in the overall population. Recently, marginal mean regression modeling procedures for zero-inflated count outcomes have been introduced within the framework of maximum likelihood estimation of zero-inflated Poisson and negative binomial regression models. In this article, we propose marginalized mixture regression models based on two-component mixtures of non-degenerate count data distributions that provide directly interpretable estimates of exposure effects on the overall population mean of a count outcome. The models are examined using simulations and applied to two datasets, one from a double-blind dental caries incidence trial, and the other from a horticultural experiment. The finite sample performance of the proposed models are compared with each other and with marginalized zero-inflated count models, as well as ordinary Poisson and negative binomial regression.

**Electronic supplementary material:**

The online version of this article (doi:10.1186/s40488-017-0057-4) contains supplementary material, which is available to authorized users.

## Introduction

The analysis of data from populations with unexplained heterogeneity presents special challenges to researchers. When count data arise from mixtures of unobserved populations, models based on standard probability distributions are often inadequate to explain observed variability (Frühwirth-Schnatter 2005; Wedel and DeSarbo 1995). For example, in dental caries research and many other areas, proportions of observations with zero counts are often higher than expected under the Poisson or negative binomial distributions, and regression models based on these distributions may result in biased estimates and poor predictions. To account for such excess zeros, Mullahy (1986) and Lambert (1992) proposed zero-inflated Poisson (ZIP) regression. ZIP models, which employ two-component mixture distributions, hypothesize that observed counts arise from one of two latent classes within the source population: one class provides only zeros and the other produces both zero and non-zero values. However, the assumption of a model based on ‘at-risk’ and ‘not-at-risk’ latent classes may not be appropriate in some settings or may provide an inadequate fit. To model counts from multiple source populations, Wang et al. (1996) proposed multi-component Poisson mixture distributions, and their approach has been extended to other finite mixtures of non-degenerate count distributions. Despite the flexibility of finite mixtures for describing highly dispersed count data, parameters from standard mixture regression models are not directly applicable to making inferences about the overall effects of covariates on marginal means of count outcomes (Albert et al. 2014; Preisser et al. 2012). Even with the application of indirect methods of parameter estimation such as the use of post-modeling transformations, there are many instances where latent class model formulations fail to fully explain relationships between covariates and population-wide parameters.

While the importance of the marginal mean as a target of inference in the analysis of finite mixtures of counts is well established (Albert et al. 2014; Böhning et al. 1999; Lambert 1992; Preisser et al. 2012), marginally-specified mean models for finite mixtures of count distributions have only recently been proposed. Within a ZIP likelihood framework, Long et al. (2014) proposed marginalized zero-inflated Poisson (MZIP) regression, which specifies a two-part model for counts with a set of regression coefficients for the marginal mean and, to complete model specification, a second set of regression coefficients for the latent parameter defining membership in the ‘excess-zero’ class. The marginalized zero-inflated negative binomial (MZINB) model (Preisser et al. 2016) extended the MZIP model to account for overdispersion in addition to excess zeros. Todem et al. (2016) described a general representation of two-part marginalized mean count models including distributions for bounded counts, e.g., the zero-inflated beta-binomial distribution. In each case, the model is assumed to follow a two-component mixture consisting of a standard count distribution with a degenerate point-mass at zero. However, data-generating mechanisms based on mixtures of non-degenerate count distributions can often provide better fits when the data suggest that a simple degenerate point-mass is insufficiently flexible to capture the heterogeneity in the counts. This can arise, for example, when there is overdispersion in the counts that cannot be fully explained by standard count data distributions (e.g., Poisson, negative binomial) amended by excess zeros.

In this article, we seek to expand the class of marginalized mixture models for zero-inflated and other heterogeneous count data to allow for greater model choice with maximum likelihood estimation, when there is interest in evaluating the effects of exposures on the overall mean count. For counts having unexplained heterogeneity, we extend the degenerate component of marginalized zero-inflated models to standard count distributions for more flexible modeling of the marginal mean. Our motivation comes from a randomized double-blind caries incidence trial conducted between 1988 and 1992 in Lanarkshire, Scotland, to compare the anti-caries efficacy of three toothpaste formulations in 4294 children ages 11–12 (Stephen et al. 1994). The outcome variable of interest was the number of new decayed, missing and filled surfaces (DMFS) two years following the baseline dental exam. Caries development is a complex process, which depends upon multiple biological and environmental factors; moreover, the clinical manifestation of disease is influenced by preventive care and restorative dental treatment decisions. For this reason, standard count models such as Poisson and negative binomial regression may not adequately account for heterogeneity in the DMFS counts. We consider marginalized, two-component finite mixture models to obtain direct inference about the relationship between toothpaste formulation and the marginal mean caries count in the trial population. “Methods and Results” section reviews traditional and marginalized zero-inflated count regression models, while “Models for mixtures of non-degenerate count distributions” section discusses traditional finite mixture regression models and proposes marginalized two-component count regression models involving mixtures of non-degenerate distributions. Simulation studies and two applications of the proposed models including the Lanarkshire caries trial are discussed in “Simulation study & Applications” sections, respectively. Concluding remarks follow in “Discussion and conclusions” section.

## Methods and Results

### Zero-inflated Poisson and negative binomial models

Traditional zero-inflated models assume that counts arise from a two-component mixture of a standard count distribution with a distribution degenerate at zero. Under such models, counts are generated either from a ‘non-susceptible’ or ‘perfect’ state that always gives zeros, or from a ‘susceptible’, ‘imperfect’ state that produces both zero and positive counts according to a standard count data distribution. Lambert (1992) introduced the zero-inflated Poisson (ZIP) regression model and applied it for modeling defects in manufacturing processes, where defects are assumed coming from a ‘perfect’ state with a probability *π* or an ‘imperfect’ state with a probability 1−*π*. While counts from the ‘perfect’, ‘no-defect’ state are always zero, those from the ‘imperfect’ state follow a Poisson distribution. The probability mass function (pmf) of a random variable having a ZIP or zero-inflated negative binomial (ZINB) distribution can be written as

1 $$\begin{array}{@{}rcl@{}}  Pr(Y_{i}=k)=\pi_{i} I(k=0)+(1-\pi_{i})g(k|{\boldsymbol{\theta}}_{i}), k=0,1,2,\ldots, \end{array} $$

where the mixing parameter *π*_*i*_ is interpreted as the probability of a count being from the ‘non-susceptible’ or ‘not-at-risk’ latent class, *I*(*T*) is an indicator variable taking 1 when *T* is true, and 0 when *T* is false; *g* is a Poisson or negative binomial mass function, and ***θ***_*i*_ is the vector of parameters in *g*. When *g* is the Poisson mass function, ***θ***_*i*_ is equal to the mean *μ*_*i*_ of the distribution, and for a negative binomial probability mass function *g*, ***θ***_*i*_=(*μ*_*i*_,*α*), where *μ*_*i*_ is the mean of the distribution and *α* is the dispersion parameter. In this paper, we will use the following parameterization for the probability mass function of a negative binomial distribution with mean *μ* and dispersion parameter *α*.

$$\begin{array}{@{}rcl@{}} f(y|\mu,\alpha) &=& \frac{\Gamma(y + \alpha)}{y! \Gamma(\alpha)} \left(\frac{\alpha}{\alpha+\mu}\right)^{\alpha} \left(\frac{\mu}{\alpha+\mu}\right)^{y}, \text{where}\, y = 0, 1, \ldots. \end{array} $$

In zero-inflated count models, the logit and the log link functions are typically specified for the mixing probability *π*_*i*_ and the mean of the assumed standard distribution *μ*_*i*_, respectively, as

$$\begin{array}{@{}rcl@{}} logit(\pi_{i}) &=& \mathbf{w}'_{i}{\boldsymbol{\gamma}} \quad \text{and} \quad log(\mu_{i}) = \mathbf{x}'_{i} {\boldsymbol{\xi}}, \end{array} $$

where **w**_*i*_ and **x**_*i*_ are *q*×1 and *p*×1 vectors of covariates for the *i* ^*th*^ subject, and ***γ***=(*γ*_1_,*γ*_2_,…,*γ*_*q*_)^′^ and ***ξ***=(*ξ*_1_,*ξ*_2_,…,*ξ*_*p*_)^′^ are regression parameters. For *n* independent observations, the ZIP likelihood function is

$$\begin{array}{@{}rcl@{}} L({\boldsymbol{\xi}}, {\boldsymbol{\gamma}}|\mathbf{y}) &=& \prod_{i=1}^{n}\lbrace 1+e^{\mathbf{w}'_{i}{\boldsymbol{\gamma}}}\rbrace^{-1} \left \{ e^{\mathbf{w}'_{i}{\boldsymbol{\gamma}}}+e^{-\exp(\mathbf{x}'_{i}{\boldsymbol{\xi}})}\right \}^{I(y_{i}=0)}\left \{ \frac {e^{-\exp(\mathbf{x}'_{i}{\boldsymbol{\xi}})}e^{\mathbf{x}'_{i}{\boldsymbol{\xi}} y_{i}}}{y_{i}!} \right \}^{I(y_{i}>0)}  \end{array} $$

The corresponding likelihood function for the ZINB model can be written as

$$\begin{array}{@{}rcl@{}} L({\boldsymbol{\xi}}, {\boldsymbol{\gamma}}, \alpha|\mathbf{y}) &=& \prod_{i=1}^{n}\lbrace 1+e^{\mathbf{w}'_{i}{\boldsymbol{\gamma}}}\rbrace^{-1} \left \{ e^{\mathbf{w}'_{i}{\boldsymbol{\gamma}}}+\left(\frac{\alpha}{\alpha+e^{\mathbf{x}'_{i}{\boldsymbol{\xi}}}}\right)^{\alpha}\right \}^{I(y_{i}=0)}  \\ && \times \prod_{i=1}^{n}\left \{ \frac{\Gamma(y_{i} + \alpha)}{y_{i}! \Gamma(\alpha)}\left(\frac{\alpha}{\alpha+e^{\mathbf{x}'_{i}{\boldsymbol{\xi}}}}\right)^{\alpha}\left(\frac{e^{\mathbf{x}'_{i}{\boldsymbol{\xi}}}}{\alpha+e^{\mathbf{x}'_{i}{\boldsymbol{\xi}}}}\right)^{y_{i}} \right \}^{I(y_{i}>0)}  \end{array} $$

Since interpretations of parameters ***γ*** and ***ξ*** in ZIP and ZINB models apply to the two latent subpopulations, they do not directly describe the overall population mean. Although the overall mean, *E*(*Y*_*i*_)=*ν*_*i*_, for *i* ^*th*^ subject could be estimated from such models by

$$\begin{array}{@{}rcl@{}} \nu_{i} &=& \frac{e^{\mathbf{x}'_{i}{\boldsymbol{\xi}}}}{1+e^{\mathbf{w}'_{i}{\boldsymbol{\gamma}}}}  \end{array} $$

and transformations such as the delta method could be applied to estimate the corresponding variance, it is not always easy to understand the behavior of *ν*_*i*_. In particular, determining the overall effects of an exposure variable on incidence density ratios is challenging especially when the linear predictors from both the mixing proportions and the Poisson mean model contain the exposure variable (Long et al. 2014).

### Marginalized ZIP and ZINB models

To estimate the overall effects of covariates on the population mean, marginalized zero-inflated Poisson (Long et al. 2014) and marginalized zero-inflated negative binomial (Preisser et al. 2016) models specify parameters for the probability of being an excess zero (i.e., *π*_*i*_) and the marginal mean *ν*_*i*_=*E*(*y*_*i*_)=(1−*π*_*i*_)*μ*_*i*_ as

$$\begin{array}{@{}rcl@{}} logit(\pi_{i}) = \mathbf{w}'_{i}{\boldsymbol{\gamma}} \quad \text{and} \quad log(v_{i}) &=& \mathbf{x}'_{i} {\boldsymbol{\beta}},  \end{array} $$

where ***β***=(*β*_1_,*β*_2_,…,*β*_*p*_) is a vector of regression parameters for *ν*_*i*_ that have the same interpretations for the effects of exposures on the marginal mean as in Poisson and negative binomial regression, whereas the parameters in ***γ*** have the same latent class interpretations for zero-inflation as in ZIP and ZINB models. The MZIP and MZINB likelihood functions are obtained by replacing *μ*_*i*_ by *ν*_*i*_/(1−*π*_*i*_) in the ZIP and ZINB likelihoods, respectively.

The next section introduces methods of estimating regression parameters for the overall population mean of heterogeneous counts generated from non-degenerate mixture distributions. With the aim of expanding the pool of two-part marginalized models for counts, special consideration is given to data generating mechanisms based on mixtures of two Poissons and a negative binomial with a Poisson distribution.

### Finite mixture models

Finite mixture distributions have been used to model counts obtained from heterogeneous populations (Wang et al. 1996; Schlattmann 2009; Morgan et al. 2014). In the general finite mixture model, the source population is assumed to be a partition of *m*≥2 latent subpopulations; with a probability *π*_*ij*_, the count random variable *Y*_*i*_ corresponding to the *i* ^*th*^ individual takes a value from the *j* ^*th*^ subpopulation according to a distribution specific to the subpopulation. An *m*-component mixture distribution can be defined as (Frühwirth-Schnatter 2005; Wedel and DeSarbo 1995)

$$\begin{array}{@{}rcl@{}} Pr(Y_{i}=y_{i}|\pi,{\boldsymbol{\theta}}_{i}) &=& \sum_{j=1}^{m} \pi_{j}f_{j}(y_{i}|{\boldsymbol{\theta}}_{ij})  \end{array} $$

where the components *f*_1_, *f*_2_, …, *f*_*m*_ are probability mass functions of known distributions, ***θ***_*i*_=(***θ***_*i*1_,…,***θ***_*im*_)^′^ where ***θ***_*ij*_ is the vector of parameters in *f*_*j*_, and ***π***=(*π*_1_,*π*_2_…,*π*_*m*_)^′^ is a vector of mixing probabilities with 0≤*π*_*j*_≤1 and $\sum _{j=1}^{m} \pi _{j} =1$. While the mixture distribution for zero-inflated counts in equation (1) allows mixing probabilities to vary across individuals, conventional finite mixture models assume a constant probability, *π*_*j*_, corresponding to the *j* ^*th*^ subpopulation and impose heterogeneity through *f*_*j*_(*y*_*i*_|***θ***_*ij*_).

The Poisson mixture distribution, where

$$\begin{array}{@{}rcl@{}} f_{j}(y_{i}|\mu_{ij})= \frac{e^{-\mu_{ij}}\mu_{ij}^{y_{i}}}{y_{i}!} \end{array} $$

with *μ*_*ij*_ being the mean of the *j* ^*th*^ component distribution, is a popular finite mixture model for count data. In Poisson mixture regression, the mean *μ*_*ij*_,*j*=1,…,*m*, is modeled as a function of covariates using the log link. Wang et al. (1996) discuss that such models are identifiable for full rank design matrices. While finite mixture models enable flexible modeling of counts from heterogeneous populations, their parameters have latent class interpretations. Such coefficients do not directly provide inferences regarding the effects of covariates on the overall population mean (Min and Agresti 2005; Roeder et al. 1999).

For *m*=2, the pmf of a random variable with a Poisson-Poisson mixture distribution can be written as

$$\begin{array}{@{}rcl@{}}  f(y_{i}|\pi, \mu_{1i}, \mu_{2i}) &=& \pi f_{P1}(y_{i}|\mu_{1i})+(1-\pi)f_{P2}(y_{i}|\mu_{2i})  \end{array} $$

where *π* is a mixing probability, and *f*_*P*1_ and *f*_*P*2_ are Poisson mass functions with corresponding mean parameters *μ*_1*i*_ and *μ*_2*i*_. Similarly, a negative binomial-Poisson random variable has a pmf given by

2 $$\begin{array}{@{}rcl@{}}  f(y_{i}|\pi_{i}, \mu_{1i}, \mu_{2i}, \alpha) = \pi f_{P}(y_{i}|\mu_{1i})+(1-\pi)f_{NB}(y_{i}|\mu_{2i},\alpha). \end{array} $$

In Eq. (2), *f*_*P*_ is a Poisson pmf with mean parameter *μ*_1*i*_ and *f*_*NB*_ a negative binomial pmf with mean and dispersion parameters *μ*_2*i*_ and *α*, respectively. The marginal mean, *ν*_*i*_, of a random variable *Y*_*i*_ having either of the two mixture distributions can be written as

3 $$\begin{array}{@{}rcl@{}}  \nu_{i} =\pi \mu_{1i}+(1-\pi) \mu_{2i}. \end{array} $$

In traditional finite mixture models, separate regression equations are specified for the mean of each component of the mixture. In general, *ν*_*i*_ depends upon a complicated function of the regression coefficients from the components. In the next section, new marginalized models are specified for direct inference regarding the effects of covariates on *ν*_*i*_.

### Marginalized finite mixture models

Solving for *μ*_2*i*_ in Eq. (3) gives

4 $$\begin{array}{@{}rcl@{}}  \mu_{2i} = \frac{\nu_{i}-\pi \mu_{1i}}{1-\pi}. \end{array} $$

To estimate a model for *ν*_*i*_, the likelihood functions of Poisson-Poisson and negative binomial-Poisson mixture models can be written as functions of *ν*_*i*_ by replacing *μ*_2*i*_ by the linear function of the marginal mean in Eq. (4). Thus, marginalized Poisson-Poisson (MPois-Pois) and negative binomial-Poisson (MNB-Pois) pmfs can be written as in Eqs. (5) and (6), respectively:

5 $$\begin{array}{@{}rcl@{}}{}  f_{MPP}(y_{i}|\pi,\mu_{1i},\nu_{i}) = \pi \frac{e^{-\mu_{1i}}\mu_{1i}^{y_{i}}}{y_{i}!}+(1-\pi) \frac{e^{-\frac{\nu_{i}-\pi\mu_{1i}}{1-\pi}}\left[\frac{\nu_{i}-\pi\mu_{1i}}{1-\pi}\right]^{y_{i}}}{y_{i}!} \end{array} $$

6 $${}  f_{NBP}(y_{i}|\pi,\alpha,\mu_{1i},\nu_{i}) = \pi \frac{e^{-\mu_{1i}}\mu_{1i}^{y_{i}}}{y_{i}!}+(1-\pi)\frac{\Gamma(y_{i} + \alpha)}{y_{i}! \Gamma(\alpha)} \left(\frac{\alpha}{\alpha+\frac{\nu_{i}-\pi\mu_{1i}}{1-\pi}}\right)^{\alpha} \left(\frac{\frac{\nu_{i}-\pi\mu_{1i}}{1-\pi}}{\alpha+\frac{\nu_{i}-\pi\mu_{1i}}{1-\pi}}\right)^{y_{i}}  $$

The MPois-Pois model is defined through Eq. (5) with specification of generalized linear models in (7) for the relationship of covariates to *ν*_*i*_ and *μ*_1*i*_,

7 $$\begin{array}{@{}rcl@{}}  log(\nu_{i})&= &\mathbf{x}'_{i}{\boldsymbol{\beta}}\\  log(\mu_{1i})&=& \mathbf{z}'_{i}{\boldsymbol{\xi}}\\  logit(\pi)&=& \rho  \end{array} $$

where **x**_*i*_ and **z**_*i*_ are vectors of covariates and ***β*** and ***ξ*** are corresponding vectors of regression coefficients, and −*∞*<*ρ*<*∞* is a constant. Although ***ξ*** and *ρ* are considered nuisance parameters that are not of primary interest, they need to be modeled to facilitate maximum likelihood estimation of ***β*** in the marginal mean model. The logarithm of *μ*_1*i*_ is modeled by using a linear predictor that involves covariates as in standard finite mixture Poisson models. The mixing parameter *π* is modeled as a constant using the logit link to guarantee that its estimate is between 0 and 1.

A common model specification is **x**_*i*_=**z**_*i*_ such that ***β*** and ***ξ*** are *p*×1 vectors of parameters. However, the covariates that are included in modeling *ν*_*i*_ and *μ*_1*i*_ may be different. As the main interest is in ***β***, a reduced set of covariates **z**_*i*_ may be considered when it is necessary for computational tractability. Alternatively, a shared-parameter model (Preisser et al. 2016) may be used to incorporate a large number of covariates with relatively few parameters.

The MNB-Pois model defined through Eqs. (6) and (7) also requires estimation of the dispersion parameter *α* via a model specified as

8 $$\begin{array}{@{}rcl@{}}  log(\alpha) = -\tau. \end{array} $$

The link functions in Eqs. (7) and (8) correspond to *ν*_*i*_>0, *μ*_1*i*_>0, 0<*π*<1 and *α*>0.

For *n* independent count random variables *Y*_1_,*Y*_2_,…,*Y*_*n*_ with corresponding realizations *y*_1_,*y*_2_,…,*y*_*n*_, the likelihood function for MPois-Pois models is given by Eq. (9).

9 $$  L(\rho, {\boldsymbol{\beta}}, {\boldsymbol{\xi}}|\mathbf{y}) = \prod_{i=0}^{n} \frac{1}{(1+e^{\rho})y_{i}!} \left \{e^{\rho}\exp(-e^{\mathbf{z}'_{i}{\boldsymbol{\xi}}})e^{\mathbf{z}'_{i}{\boldsymbol{\xi}} y_{i}}+e^{-\eta(\rho, {\boldsymbol{\beta}}, {\boldsymbol{\xi}};\mathbf{x}_{i},\mathbf{z}_{i})} \eta(\rho, {\boldsymbol{\beta}}, {\boldsymbol{\xi}};\mathbf{x}_{i},\mathbf{z}_{i})^{y_{i}} \right \}  $$

with

10 $$\begin{array}{@{}rcl@{}}  \eta(\rho, {\boldsymbol{\beta}}, {\boldsymbol{\xi}};\mathbf{x}_{i},\mathbf{z}_{i})= e^{\mathbf{x}'_{i}{\boldsymbol{\beta}}}(1+e^{\rho})-e^{\rho}e^{\mathbf{z}'_{i}{\boldsymbol{\xi}}}. \end{array} $$

Similarly, the likelihood function for the MNB-Pois model can be specified as

$$\begin{array}{@{}rcl@{}} L(\rho, \tau,{\boldsymbol{\beta}}, {\boldsymbol{\xi}}|\mathbf{y}) & = & \prod_{i=0}^{n} \bigg \{ \frac{\Gamma(y_{i}+e^{-\tau})}{(1+e^{\rho})\Gamma(y_{i}+1)\Gamma(e^{-\tau})}\left(\frac{e^{-\tau}}{e^{-\tau}+\eta(\rho, {\boldsymbol{\beta}}, {\boldsymbol{\xi}};\mathbf{x}_{i},\mathbf{z}_{i})}\right)^{e^{-\tau}}  \\ & & \times \left(\frac{\eta(\rho, {\boldsymbol{\beta}}, {\boldsymbol{\xi}};\mathbf{x}_{i},\mathbf{z}_{i})}{e^{-\tau}+\eta(\rho, {\boldsymbol{\beta}}, {\boldsymbol{\xi}};\mathbf{x}_{i},\mathbf{z}_{i})}\right)^{y_{i}} \bigg \} + \prod_{i=0}^{n} \frac{e^{\rho}\exp(-e^{\mathbf{z}'_{i}{\boldsymbol{\xi}}})e^{\mathbf{z}'_{i}{\boldsymbol{\xi}} y_{i}}}{(1+e^{\rho})y_{i}!}  \end{array} $$

where *η*(*ρ*,***β***,***ξ***;**x**_*i*_,**z**_*i*_) has the same expression as in Eq. (10). With carefully chosen starting parameter values, marginalized finite mixture models can be fitted using quasi-Newton optimization. Guidance for specifying starting values and use of SAS Proc NLMIXED for fitting the proposed models is presented as Additional file 1 (Benecha et al., 2017) along with further discussion of connections between the models in “Marginalized ZIP and ZINB models” and “Marginalized finite mixture models” sections.

Finally, with respect to mixture Eq. (2), solving for *μ*_1*i*_ in (3) gives

11 $$\begin{array}{@{}rcl@{}}  \mu_{1i}=\frac{\nu_{i}-(1-\pi) \mu_{2i}}{\pi}. \end{array} $$

Inserting this expression for *μ*_1*i*_ in the standard mixture likelihood function based on Eq. (2) gives a likelihood function for a model that is different from MNB-Pois. The alternative model, which marginalizes over the Poisson part versus MNB-Pois that marginalizes over the NB part, is not considered owing to unresolved computational issues in the applications.

### Simulation study

Simulation studies were performed to examine the properties of MPois-Pois and MNB-Pois models for various sample sizes. Counts from these models were generated from the probability mass functions in Eqs. (5) and (6), where *π*, *μ*_1*i*_, *ν*_*i*_ and *α* are determined from

$$\begin{array}{@{}rcl@{}} log(\nu_{i})&=& \mathbf{x}'_{i}{\boldsymbol{\beta}}= \beta_{0}+\beta_{1}x_{1i}+\beta_{2}x_{2i}+\beta_{3}x_{3i}  \\ log(\mu_{1i})&=& \mathbf{z}'_{i}{\boldsymbol{\xi}}= \xi_{0}+\xi_{1}x_{1i}+\xi_{2}x_{2i}+\xi_{3}x_{3i}  \\ logit(\pi)&=& \rho,  \\ log(\alpha)&=& -\tau  \end{array} $$

with **x**_*i*_=**z**_*i*_ and *x*_1*i*_∼Poisson(2)/3, *x*_2*i*_∼exp(1), *x*_3*i*_∼Benoulli(0.4), *β*_0_=1.5, *β*_1_=−0.1, *β*_2_=−0.2, *β*_3_=0.5, *ξ*_0_=1.5, *ξ*_1_=−0.5, *ξ*_2_=−0.5, *ξ*_3_=1, *ρ*=−0.4 and *τ*=−0.5. Using these specifications, samples of sizes 100, 200, 500 and 1000 were generated corresponding to MPois-Pois and MNB-Pois models. Poisson and negative binomial (NB) regression and four marginalized count models, namely MZIP, MZINB, MPois-Pois and MNB-Pois, were then fitted to the data, where each simulation was repeated 10,000 times. To estimate Type I error rates of testing *H*_0_:*β*_1_=0 vs *H*_1_:*β*_1_≠0, all the simulations were repeated by generating data using *β*_1_=0, but keeping all the remaining parameter and covariate values the same as described previously. For each of the six models, the Type I error rates were calculated among converged model fits as the proportion of *p*-values from two-sided Wald tests that were less than 0.05.

For MPois-Pois generated data, estimates of *β*_1_, *β*_2_ and *β*_3_ had low biases for all models and all sample sizes (Table 1). The MPois-Pois model had Type I error rates for *β*_1_ close to 0.05, while the remaining models tended to over-estimate the error rates (Table 2). The MPois-Pois model estimated coverages of 95% confidence intervals for *β*_1_, *β*_2_ and *β*_3_ that were close to the nominal value (Table 3). Whereas NB, MZINB and MNB-Pois models tended to have only slight undercoverage, Poisson and MZIP had coverage ranging from 88 to 92%. Convergence rates for MPois-Pois simulation scenarios ranged from 96.2 to 99.3%, while convergence rates ranged from 88.0 to 90.2% for MNB-Pois, from 75.9 to 98.4% for MZIP, and from 72.0 to 96.6% for the MZINB models. Convergence was 100% for Poisson and NB regression for all sample sizes.

**Table 1:** Percent relative median biases of estimates of *β*_1_, *β*_2_ and *β*_3_ from marginalized mixture models fitted to data generated from the MPois-Pois model with 10,000 replications

Sample size	Parameter	Poisson	MZIP	MPois-Pois	NB	MZINB	MNB-Pois
100	*β*_1_	2.03	1.40	−2.04	2.18	0.97	0.56
	*β*_2_	0.77	−3.11	0.08	1.00	−3.45	1.54
	*β*_3_	−0.30	−0.61	−0.70	−0.26	−0.74	−0.33
200	*β*_1_	0.97	1.70	−0.68	1.38	1.89	1.34
	*β*_2_	−0.02	−2.64	−0.69	0.08	−2.65	0.62
	*β*_3_	0.15	−0.43	−0.29	−0.09	−0.41	0.06
500	*β*_1_	−0.68	−0.36	−0.87	−0.79	−1.18	0.07
	*β*_2_	0.04	−1.51	0.11	0.09	−1.44	0.78
	*β*_3_	0.08	−0.16	−0.14	0.05	−0.11	0.19
1000	*β*_1_	−0.14	−0.37	−0.40	−0.07	−0.64	0.43
	*β*_2_	0.48	−1.43	0.27	0.50	−0.91	0.88
	*β*_3_	0.09	−0.08	0.06	0.07	−0.07	0.22

**Table 2:** Type I error rates for the estimate of *β*_1_ from marginalized models fitted to data generated from the MPois-Pois model with 10,000 replications

Sample size	Poisson	MZIP	MPois-Pois	NB	MZINB	MNB-Pois
100	0.127	0.102	0.068	0.077	0.070	0.073
200	0.131	0.106	0.067	0.077	0.072	0.069
500	0.135	0.112	0.060	0.079	0.073	0.065
1000	0.134	0.112	0.054	0.072	0.066	0.061

**Table 3:** Coverages of 95% confidence intervals for estimates of *β*_1_, *β*_2_ and *β*_3_ from marginalized models fitted to data generated from the MPois-Pois model with 10,000 replications

Sample size	Parameter	Poisson	MZIP	MPois-Pois	NB	MZINB	MNB-Pois
100	*β*_1_	89.9	91.3	93.7	93.3	93.4	93.8
	*β*_2_	89.2	90.9	93.2	92.4	92.8	92.9
	*β*_3_	91.8	92.9	95.2	94.9	94.7	95.1
200	*β*_1_	89.4	91.2	94.1	93.6	93.8	94.1
	*β*_2_	88.9	90.9	93.3	92.2	92.9	93.2
	*β*_3_	91.4	92.6	95.1	95.1	94.9	95.2
500	*β*_1_	88.9	90.7	94.1	92.9	93.5	93.9
	*β*_2_	88.6	90.5	94.4	92.0	93.1	93.9
	*β*_3_	91.0	92.0	94.9	95.1	94.8	94.9
1000	*β*_1_	89.3	90.9	94.7	93.5	93.9	94.4
	*β*_2_	88.5	90.8	94.7	92.1	93.1	93.8
	*β*_3_	91.1	92.1	95.0	95.0	95.0	94.9

When the data are generated from the MNB-Pois model, the MNB-Pois model had low percent relative median biases for *β*_1_, *β*_2_ and *β*_3_, and the biases appear to decrease as sample sizes increase (Table 4). The corresponding estimates from the Poisson, NB and MZINB models also have low biases, but those from MPois-Pois and MZIP models are generally higher. In addition, the performance of the true MNB-Pois model with regard to Type I error rates (for *β*_1_) and coverages of 95% confidence intervals (for *β*_1_, *β*_2_ and *β*_3_) is superior to Poisson, MZIP and MPois-Pois models at all sample sizes (Tables 5 and 6, respectively) and has better performance than NB and MZINB for the sample sizes of 500 and 1000. Over 96% of MNB-Pois models converged for sample sizes of 200 or more, with 91% convergence for sample size of 100. Coverage ranged from 97.4 to 100% for MZIP, from 92.0 to 99.4% for the MPois-Pois models, from 85.3 to 91.4% for MZINB models and rates were 100% for Poisson and NB regression. Overall, the simulation results indicate that when the true model is specified, MPois-Pois or MNB-Pois models estimate marginal mean regression parameters with small biases, Type I errors close to the assumed rate and coverages of 95% confidence intervals near 95% for sample sizes of 200 or greater.

**Table 4:** Percent relative median biases of estimates of *β*_1_, *β*_2_ and *β*_3_ from marginalized mixture models fitted to data generated from the MNB-Pois model with 10,000 replications

Sample size	Parameter	Poisson	MZIP	MPois-Pois	NB	MZINB	MNB-Pois
100	*β*_1_	6.94	23.51	6.80	10.48	13.72	11.95
	*β*_2_	2.98	7.95	4.00	5.26	1.89	4.44
	*β*_3_	0.01	1.40	−4.35	0.80	0.88	−0.25
200	*β*_1_	5.27	20.12	−14.85	5.45	7.41	4.57
	*β*_2_	1.45	5.11	−1.12	2.49	0.07	2.02
	*β*_3_	0.18	1.49	−5.44	0.38	0.36	0.33
500	*β*_1_	0.57	11.79	−29.97	1.31	0.73	−0.75
	*β*_2_	0.66	2.81	−3.90	1.18	0.14	0.62
	*β*_3_	0.39	1.52	−7.66	0.59	0.61	0.46
1000	*β*_1_	1.19	10.34	−34.68	1.92	2.39	0.00
	*β*_2_	0.79	2.63	−4.75	1.00	0.39	0.87
	*β*_3_	−0.01	0.97	−10.13	0.03	−0.01	−0.19

**Table 5:** Type I error rates for the estimate of *β*_1_ from marginalized models fitted to data generated from the MNB-Pois model with 10,000 replications

Sample size	Poisson	MZIP	MPois-Pois	NB	MZINB	MNB-Pois
100	0.325	0.271	0.262	0.084	0.079	0.103
200	0.334	0.272	0.255	0.079	0.073	0.064
500	0.341	0.273	0.232	0.081	0.074	0.053
1000	0.340	0.273	0.240	0.076	0.072	0.049

**Table 6:** Coverages of 95% confidence intervals for estimates of *β*_1_, *β*_2_ and *β*_3_ from marginalized models fitted to data generated from the MNB-Pois model with 10,000 replications

Sample size	Parameter	Poisson	MZIP	MPois-Pois	NB	MZINB	MNB-Pois
100	*β*_1_	72.3	77.4	76.9	92.5	92.4	89.7
	*β*_2_	74.0	79.6	77.8	90.8	91.8	89.6
	*β*_3_	74.4	79.4	83.0	94.0	93.7	92.0
200	*β*_1_	71.6	77.6	78.1	92.2	92.3	93.0
	*β*_2_	72.8	79.1	78.9	91.0	91.8	92.7
	*β*_3_	74.1	80.0	83.9	94.4	94.0	93.5
500	*β*_1_	71.2	77.0	78.1	92.1	92.2	94.2
	*β*_2_	72.3	78.6	80.8	90.5	91.3	94.5
	*β*_3_	73.6	79.7	80.2	94.3	93.9	94.5
1000	*β*_1_	71.7	77.5	76.2	92.7	93.1	95.0
	*β*_2_	73.0	78.9	81.5	90.2	91.6	95.0
	*β*_3_	74.1	80.7	71.6	94.6	94.6	95.3

## Applications

### A caries incidence trial

**Fig. 1:**
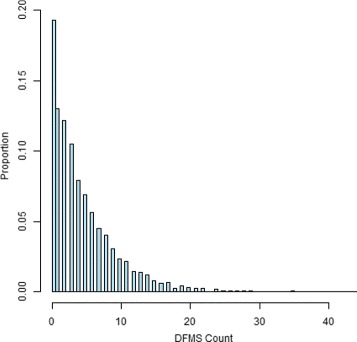
Histogram of two-year DMFS increment for 3412 children ages 11–12 from a dental caries incidence trial conducted in Lanarkshire, Scotland between 1988 and 1992

The methods described in this article were applied to the Lanarkshire caries incidence trial introduced in “Introduction” section. A total of 4294 children ages 11–12 were randomized to either sodium fluoride (NaF), sodium fluoride plus sodium trimetaphosphate (NaFTMP) or sodium monofluorophosphate (SMFP) toothpaste formulations and dental exams were performed at baseline and after 1, 2 and 3 years. The analysis was based on 3412 children followed up until year 2 and the response variable of interest was the number of new decayed, missing and filled surfaces (DMFS). Let *NaF*=1 if the child was given sodium fluoride and 0 otherwise and let *NaFTMP*=1 if the child was randomized to the NaFTMP group and 0 otherwise; children in the SMFP group make up the reference treatment category (*NaF*=*NaFTMP*=0). In addition to treatment allocation, baseline caries (bc: 1= high, 0 = low) and baseline calculus (calc:1=yes, 0= no) were considered as explanatory variables. High baseline caries values correspond to at least one decayed, missing or filled anterior tooth or premolar, and a baseline calculus value of ‘1’ refers to the existence of calcified deposits on the teeth formed by the continuous presence of dental plaque (Stephen et al. 1994; Preisser et al. 2014). An important feature of the data is the large number of zero counts in the outcome variable, as 658 (19.28%) of the 3412 children had zero DMFS counts (Fig. 1). Since the percentage of zeros is high, two-part marginalized models may provide less biased estimates and better model fits than one-part generalized linear models.

**Fig. 2:**
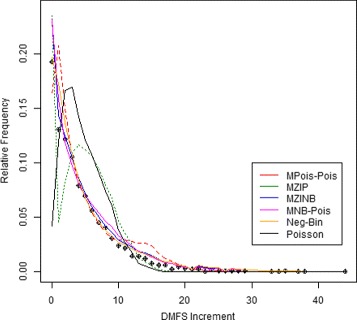
Observed (*circles*) and predicted relative frequencies of two-year DMFS increment for children in the Lanarkshire caries trial

Poisson, NB, MZIP, MZINB, MPois-Pois and MNB-Pois models were applied to compare the efficacy of the toothpaste formulations with respect to the marginal mean DMFS count. In the two-part models, the four binary covariates defined above were included in each model part. The three best models were NB, MZINB and MNB-Pois, which produced fitted values that best matched the observed distribution of DMFS counts (Fig. 2) and have the lowest AICs (Table 7). On the other hand, Poisson, MZIP and MPois-Pois models, which did not directly account for overdispersion, had poor fits and gave standard errors of regression coefficients for the marginal mean model that were too small. The MNB-Pois model gave the best fit (lowest AIC) while its marginal mean model parameter estimates and standard errors were similar in value to those of the next best fitting model, MZINB.

**Table 7:** Marginalized count regression model estimates (est) and standard errors (SE) for the Lanarkshire caries trial

	Poisson		NB		MZIP		MZINB		MPois-Pois		MNB-Pois	
Variable	est	se	est	se	est	se	est	se	est	se	est	se
Marginal mean model												
Intercept	1.216	0.017	1.201	0.035	1.234	0.022	1.206	0.034	1.309	0.029	1.207	0.035
bc	0.758	0.017	0.757	0.036	0.765	0.023	0.757	0.035	0.663	0.026	0.757	0.035
calc	−0.204	0.020	−0.189	0.040	−0.211	0.025	−0.195	0.039	−0.263	0.031	−0.199	0.039
NaF	−0.072	0.018	−0.056	0.039	−0.098	0.023	−0.060	0.038	−0.144	0.028	−0.060	0.038
NaFTMP	−0.052	0.022	−0.022	0.048	−0.104	0.029	−0.034	0.047	−0.062	0.035	−0.033	0.047
					Zero-inflation model				Latent class mean model			
Intercept					−1.280	0.079	−2.257	0.290	2.131	0.031	−1.425	0.513
bc					−1.309	0.113	−2.911	1.571	0.512	0.027	3.539	0.500
calc					0.129	0.095	−0.149	0.273	−0.249	0.031	0.022	0.173
NaF					0.208	0.096	0.419	0.307	−0.152	0.029	−0.013	0.188
NaFTMP					0.289	0.119	0.545	0.363	−0.081	0.037	−0.307	0.201
Mixing probability model estimates^a^												
*ρ*									−0.753	0.055	−1.818	0.170
*π*									0.320		0.140	
Dispersion estimates^b^												
*τ*											−0.328	0.042
*ϕ*			0.806	0.028			0.639	0.038			0.720	
Model fit statistics												
−2loglik	22706		17249		20410		17189		18048		17169	
AIC	22716		17261		20430		17211		18070		17193	

^a^Note that *π*=1/(1+*e* ^−*ρ*^)

^b^also, *τ*= log*ϕ*

Based on the MNB-Pois model, the estimated caries incidence density ratio for the children who used the NaF toothpaste formulation versus children with the same baseline status of caries and calculus who used SMFP was exp(−0.060)=0.942 (95% CI: 0.874, 1.015; Table 8). The estimated caries incidence density ratio for the NaFTMP toothpaste relative to SMFP was exp(−0.033)=0.968 (95% CI: 0.882, 1.062). Thus, children in the NaF and NaFTMP groups had a decrease in the marginal mean DMFS count by 5.8 and 3.2%, respectively, compared to children with the same baseline characteristics who were assigned to the SMFP group. However, the associations are not statistically significant since the confidence intervals of the two incidence density ratios include 1. Conversely, inappropriate selection of the Poisson, MZIP or MPois-Pois models would have resulted in the potentially misleading conclusion that toothpaste formulation with sodium fluoride significantly reduces two-year incident caries relative to SMFP in this population of children.

**Table 8:** Estimated log-likelihood, AIC and incidence density ratios (95% CI) comparing NaF and NaFTMP with SMFP in the Lanakshire trial, based on four marginalized models

	Incidence Density Ratio (95% CI)	
Model	NaF	NaFTMP
Poisson	0.931 (0.898, 0.965)	0.949 (0.909, 0.992)
NB	0.946 (0.875, 1.022)	0.979 (0.890, 1.076)
MZIP	0.907 (0.867, 0.948)	0.902 (0.852, 0.953)
MZINB	0.942 (0.874, 1.015)	0.967 (0.881, 1.061)
MPois-Pois	0.866 (0.820, 0.915)	0.940 (0.879, 1.006)
MNB-Pois	0.942 (0.874, 1.015)	0.968 (0.882, 1.062)

### Number of roots produced by shoots of the apple cultivar *Trajan*

In a horticultural experiment, Marin et al. (1993) recorded the number of roots produced by 270 micro-propagated shoots of the columnar apple cultivar *Trajan.* During the rooting period, all shoots were maintained under identical conditions, but the shoots themselves were cultured on media containing different concentrations of the cytokinin 6-benzylaminopurine (BAP), i.e., 2.2, 4.4, 8.8, and 17.6 *μ* *M*, in growth cabinets with an 8 or 16 hour photoperiod. These data have been previously analyzed by Ridout et al.(1998, 2001) and Yang et al. (2009).

Each of the eight treatment combinations consisted of either 30 or 40 shoots, hence resulting in a total of 270 shoots. Overall, 23.7% of the root counts were zero. However, only two of 140 shoots produced under the 8 hour photoperiod were zeros whereas 62 of 130 shoots produced under the 16 h photoperiod failed to produce roots.

Five models including Poisson and NB regression were fitted with the following covariates for the marginal mean: photoperiod (taking a value of 0 if 8 h and 1 if 16 h), log(BAP concentration/2.2), and their interaction; the MZIP and MZINB models additionally included photoperiod in the logit model part for zero-inflation whereas a MPois-Pois model had a constant mixing parameter while including photoperiod in the latent mean model part. Computational issues precluded fitting the MNB-Pois model.

Among the five models, the MPois-Pois model provided the best fit having the lowest AIC with the MZINB model fitting the second best (Table 9). Based on the MPois-Pois model, under the 8 hour photoperiod, each doubling of BAP concentration (i.e., a natural log(2) change) resulted in a statistically significant 5.7% (= [ exp(log(2)×0.080)−1]×100*%*) *increase* in the number of roots produced (95% CI: 0.9%, 10.7%). Conversely, under the 16 hour photoperiod, each doubling of BAP concentration resulted in a statistically significant 9.1% (=[1− exp(0.693×−0.138)]×100*%*) *decrease* in the number of roots produced (95% CI: 0.5%, 17.1%). The 16 hour photoperiod produced about half the number of roots as the 8 hour photoperiod (Table 10).

**Table 9:** Marginalized count regression model estimates (est) and stanard errors (se) for the number of roots produced by 270 shoots of the apple cultivar *Trajan*

	Poisson		NB^a^		MZIP		MZINB^b^		MPois-Pois	
Variable	est	se	est	se	est	se	est	se	est	se
Marginal mean model										
Intercept	1.880	0.058	1.876	0.126	1.854	0.060	1.855	0.072	1.863	0.056
Photoperiod, 16h	-0.711	0.104	-0.706	0.188	-0.620	0.134	-0.618	0.152	-0.687	0.144
log(BAP/2.2)	0.069	0.042	0.073	0.092	0.092	0.042	0.091	0.052	0.080	0.034
Interaction	-0.176	0.077	-0.182	0.138	-0.258	0.078	-0.259	0.094	-0.218	0.075
					Zero-inflation model				Latent class mean model	
Intercept					-4.262	0.732	-4.381	0.827	2.142	0.051
Photoperiod, 16h					4.159	0.753	4.264	0.846	-4.238	0.552
Mixing probability model^c^										
*ρ*									0.178	0.184
*π*									0.544	
Model fit statistics										
-2loglik	1566.4		1402.1		1250.2		1236.5		1236.4	
AIC	1574.4		1412.1		1262.2		1250.5		1250.4	

^a^In the NB model, $\hat {\phi }=0.522$ (s.e. = 0.083)

^b^In the MZINB model, $\hat {\tau }=-2.662$ (s.e. 0.351) corresponding to $\hat {\phi }=e^{\hat {\tau }}=0.070.$

^c^In the MPois-Pois model, *π*=1/(1+*e* ^−*ρ*^)

**Table 10:** Model-predicted mean number of roots of the apple cultivar *Trajan* produced by the eight treatments

Treatment	No. of shoots	Observed mean	Count regression model^a^				
			Poisson	NB	MZIP	MZINB	MPP
8h + BAP 2.2	30	5.83	6.55	6.53	6.39	6.39	6.44
8h + BAP 4.4	30	7.77	6.87	6.87	6.81	6.81	6.81
8h + BAP 8.8	40	7.50	7.21	7.22	7.25	7.25	7.20
8h + BAP 17.6	40	7.15	7.56	7.60	7.73	7.72	7.60
16h + BAP 2.2	30	3.27	3.22	3.22	3.43	3.45	3.24
16h + BAP 4.4	30	2.73	2.99	2.99	3.06	3.07	2.95
16h + BAP 8.8	30	3.13	2.78	2.77	2.73	2.73	2.68
16h + BAP 17.6	40	2.45	2.58	2.57	2.43	2.43	2.43

^a^MPP = MPois-Pois model

## Discussion and conclusions

In this article, marginal means of counts with unexplained heterogeneity were modeled using two-component finite mixture distributions. Regression parameters were specified in two-part marginalized models for direct estimation of exposure effects on the overall mean count using maximum likelihood methods. Specifically, the proposed MPois-Pois and MNB-Pois mixture models provide alternative model choices to MZIP and MZINB for counts that are overdispersed or have many zeros. It may not always be clear whether a zero-inflated count model or a model based on a finite mixture of two non-degenerate components is more appropriate as Poisson and negative binomial distributions with small means can generate a large amount of zeros. In the case of dental caries, zero-inflated count regression models are sometimes used (Preisser et al. 2012) even though caries researchers question whether any child can be immune to developing caries (Mwalili et al. 2008). In many fields, use of finite mixtures of non-degenerate components may have a stronger theoretical basis than assuming a mixture of at-risk and not-at-risk latent classes. While there is sometimes interest in latent classes, researchers across many fields of inquiry are frequently interested in quantifying the effects of covariates on the overall mean count while adjusting for unexplained heterogeneity. In such cases, marginal mean regression parameters in MZIP, MZINB, MPois-Pois and MNB-Pois models have straightforward interpretations in describing overall exposure effects on count outcomes.

As described in the Additional file 1 (Benecha et al. 2017), the marginalized models proposed in this article belong to a larger class of marginalized mixture models for counts. In particular, when the mixing probability component of the model is fixed either with or without covariates, the MZIP and MZINB models may be viewed as special cases of corresponding MPois-Pois and MNB-Pois models where the Poisson component of the latter two models has a mean of zero, rendering that component degenerate. In this sense, the proposed models expand the family of two-part marginalized regression models by providing alternatives to MZIP and MZINB regression. In the absence of theoretical justification, the merit of each model in the larger class of alternative marginalized models is judged based on goodness of fit considerations. Because our main interest is in modeling marginal means of counts, model parameters that are not of primary interest are allowed to depend on covariates, or none whatsoever, to complete specification of the likelihood function. This provides for model parsimony as needed while allowing all the relevant covariates to be estimated in the marginal mean model.

A simulation study indicated that when the true model is specified, each of the proposed marginalized mixture models provides low biases, Type I errors and confidence interval coverages close to the nominal levels. As shown in additional simulation studies reported in Benecha et al. (2017), model mis-specification can result in undercoverage and inflated Type I errors. Use of empirical covariance estimation as proposed by Long et al. (2014) for MZIP models would likely improve coverage and Type I errors for large samples. In any case, assessment of model goodness-of-fit is highly recommended. Unfortunately, such assessment is often hampered by computational difficulties in fitting complex models such as MNB-Pois when the data at hand do not contain sufficient information to estimate all the model parameters. Reducing the number of covariate parameters often provides an expeditious remedy for this situation. Another advance would be to develop score tests for goodness of fit, as proposed by Ridout et al. (2001) in comparing ZIP and ZINB models, that do not require fitting the model under the alternative hypothesis.

In summary, the proposed marginalized mixture modeling framework provides a wide range of alternatives to directly estimate exposure effects on marginal means of counts generated from heterogeneous populations. The methods are fairly straightforward while requiring consideration of carefully chosen starting values and can be implemented in most statistical software. Future research could extend the marginalized count regression models to mixtures of two negative binomial distributions or to those based on Eq. (11) as an alternative to MNB-Pois, to allow the mixing probabilities to depend on covariates, and to longitudinal data.

## Additional file

Additional file 1 Marginalized Mixture Models for Count Data from Multiple Source Populations. Supplemental Material. (PDF 100 kb)
