# Supplementary material for: Marginalized mixture models for count data from multiple source populations
Source: J Stat Distrib Appl. 2017 Apr 7;4(1):3. doi: 10.1186/s40488-017-0057-4 (PMC5384970; doi:10.1186/s40488-017-0057-4)
Supplement: Additional file 1 — Marginalized Mixture Models for Count Data from Multiple Source Populations. Supplemental Material. (PDF 100 kb) [file 40488_2017_57_MOESM1_ESM.pdf]

Marginalized Mixture Models for Count Data from Multiple Source Populations

Supplemental Material

by Habtamu K. Benecha, Brian Neelon, Kimon Divaris and John S. Preisser

1 A general finite mixture model

To explicate the connection between marginalized zero-inflated count models (e.g., MZIP, MZINB) and marginalized finite mixture models (e.g., MPois-Pois, MNB-Pois), we introduce a general class of models encompassing them. An m-component mixture distribution can be defined as

$$Pr(Y_i = y_i|\pi_i, \boldsymbol{\theta}_i) \;=\; \sum_{j=1}^{m} \pi_{ij} f_j(y_i|\boldsymbol{\theta}_{ij})$$

where, unlike in section 3 of the main article, $\boldsymbol{\pi}_i = (\pi_{i1}, \pi_{i2} ..., \pi_{im})'$ is a vector of mixing probabilities that may vary across observations with $0 \leq \pi_{ij} \leq 1$ and $\sum_{j=1}^{m} \pi_{ij} = 1$. Consider the special case of $m = 2$ and a Poisson-Poisson mixture with probability mass function

$$f(y_i|\pi_i, \mu_{1i}, \mu_{2i}) \;=\; \pi_i f_{P1}(y_i|\mu_{1i}) + (1 - \pi_i) f_{P2}(y_i|\mu_{2i}),$$

where π_i is a mixing probability, and f_{P1} and f_{P2} are Poisson mass functions with corresponding mean parameters μ_{1i} and μ_{2i}. The model in its general form is:

$$\begin{aligned} log(\nu_i) &= \mathbf{x}_i'\boldsymbol{\beta} \\ log(\mu_{1i}) &= \mathbf{z}_i'\boldsymbol{\xi} \\ logit(\pi_i) &= \mathbf{w}_i'\boldsymbol{\gamma} \end{aligned} \qquad (1)$$

where $\mathbf{x}_i$, $\mathbf{z}_i$, and $\mathbf{w}_i$ are possibly different vectors of covariates, $\boldsymbol{\beta}$, $\boldsymbol{\xi}$, and $\boldsymbol{\gamma}$ are vectors of regression coefficients and $\nu_i = \pi_i \mu_{1i} + (1 - \pi_i)\mu_{2i}$. In many applications, likelihood estimation may be intractable and Bayesian methods would provide a feasible estimation approach. The purpose of this discussion is to relate model (1) to the models investigated in the main article. In particular, when $w_i = 1$ model (1) reduces to the MPP model given in equation (7), or by extension to the MNB-Pois in the case of the NB-Poisson mixture. Conversely, when $\mathbf{z}_i'\boldsymbol{\xi} \to -\infty$ for all i, then $\mu_{1i} \to 0$ and model (1) essentially reduces to the MZIP model (or MZINB in the case of the NB base distribution). This is because a Poisson distribution with mean $\mu_{1i} = 0$ is the same as a degenerate distribution that only takes 0 values.

2 Simulations: MZIP and MZINB generated counts

Simulation studies were performed to examine the properties of marginalized two-part count regression models for counts generated from MZIP and MZINB distributions. To assess

the performances of the marginalized count regression models (MZIP, MZINB, MPois-Pois, MNB-Pois) in fitting zero-inflated data, we carried out simulations by generating counts from MZINB and MZIP distributions. For these simulations and those reported in the main article, starting values for MPois-Pois and MNB-Pois were determined by using the EM-algorithm described in the section to follow.

To evaluate the performances of MPois-Pois and MNB-Pois models for zero-inflated counts, we generated data from MZINB and MZIP models and fitted MZIP, MZINB as well as the proposed marginalized models to the data. Counts were generated from the MZINB model using (2), where ν_i is the marginal mean for subject i, π_i is the excess zero probability, α is the dispersion parameter, $x_{1i} \sim$ Poisson(2)/3 , $x_{2i} \sim exp(1)$, $x_{3i} \sim$ Benoulli(0.4), $\beta_0 = 1.5$, $\beta_1 = 0.5$, $\beta_2 = -0.5$, $\beta_3 = 0.5$, $\gamma_0 = -2$, $\gamma_1 = -1$, $\gamma_2 = 1$, $\gamma_3 = -1$ and $\tau = 0$.

$$\begin{aligned} log(\nu_i) &= \beta_0 + \beta_1 x_{1i} + \beta_2 x_{2i} + \beta_3 x_{3i} \\ logit(\pi_i) &= \gamma_0 + \gamma_1 x_{1i} + \gamma_2 x_{2i} + \gamma_3 x_{3i} \\ log(\alpha) &= -\tau \end{aligned} \quad (2)$$

Similarly, counts were generated from the MZIP model in equation (3) using the same covariates as in equation (2) and with $\beta_0 = 2$, $\beta_1 = -0.5$, $\beta_2 = -0.5$, $\beta_3 = 0.5$, $\gamma_0 = -1$, $\gamma_1 = -0.5$, $\gamma_2 = 0.5$ and $\gamma_3 = -0.5$.

$$\begin{aligned} log(\nu_i) &= \beta_0 + \beta_1 x_{1i} + \beta_2 x_{2i} + \beta_3 x_{3i} \\ logit(\pi_i) &= \gamma_0 + \gamma_1 x_{1i} + \gamma_2 x_{2i} + \gamma_3 x_{3i} \end{aligned}$$

For each simulated data, MPois-Pois, MNB-Pois, MZIP and MZINB models were fitted with 10000 replications.

When the true model is MZIP, MZIP converged 99.8% of the time whereas MZINB converged from almost 90% of the time; on the other hand, MPois-Pois and MNB-Pois models suffer from poor convergence rates (Table 1). Tables 2 shows that estimates of β_1, β_2 and β_3 from MPois-Pois and MNB-Pois models have high biases compared to MZIP and MZINB. In addition, MPois-Pois and MNB-Pois models inflate the Type I error rate for β_1 (Table 3) and underestimate coverages of 95 % confidence intervals (Table 4) when fitted to data generated from the MZIP model.

With respect to the poor convergence results reported in Table 1, we note that the true MZIP model contained 8 parameters, whereas the MZINB, MPois-Pois and MNB-Pois models had 9, 9 and 10 parameters, respectively. The especially poor convergence rates for MNB-Pois is likely related to the difficulty of estimating two additional parameters from data that frequently contain insufficient information for doing so. However, MPois-Pois also had much poorer convergence rates when compared to MZINB, even though they had the same number of parameters. Included among the "non-converged" results are results with non-positive-definite Hessian matrices but that had otherwise converged to produce parameter estimates, while being unable to estimate some of the standard errors.

While the MZIP and the MZINB models have high convergence rates when the true model is MZINB, the rates range from 84.8% - 97.0% for the MPois-Pois and from 79.7% - 96.0% for the MNB-Pois models (Table 5). Table 6 shows that all the four marginalized models generally provide estimates with low bias for β_1, β_2 and β_3 when fitted to data generated from MZINB, and estimates from the MNB-Pois model have lower bias compared to those from MPois-Pois and MZIP models. The estimated Type I error rates for β_1 are close to the nominal value when the true model is fitted, but the MNB-Pois model also performs well when sample size increases (Table 7). On the other hand, the estimated Type I error rates from MPois-Pois and MZIP models are highly inflated. Table 8 shows that coverages of 95% confidence intervals for β_1, β_2 and β_3 are below 70% when MPois-Pois and MZIP models are fitted. For larger sample sizes, the MNB-Pois model provides coverages close to 95% for β_1 and β_3, but estimates for β_2 corresponding to the skewed (exponential) covariate are substantially lower than 95% for all sample sizes.

3 Finding Starting Values of Parameters

With carefully chosen starting parameter values, regression coefficients in MPois-Pois and MNB-Pois models can be estimated by the use of quasi-Newton optimization. Starting values for parameters in the models for π, μ_{1i}, and α may be obtained from standard two-component Poisson-Poisson and negative binomial-Poisson models.

Two alternative approaches are suggested for finding starting values. Following Ramaswamy *et al.* (1994) and Leisch (2004), an EM algorithm is described immediately below to find starting values for parameters ρ, $\boldsymbol{\xi}$ and τ in MNB-Pois models. A similar approach can be applied for MPois-Pois models. The EM approach was used in the simulations. An approach for finding starting values based on the use of SAS software is described further below.

3.1 Finding Starting Values with the EM-Algorithm

Consider a random variable Y_i that takes a value y_i according to the standard two-component NB-Pois mixture model in equation (3):

$$f(y_i|\pi_i, \mu_{1i}, \mu_{2i}, \alpha) = \pi f_P(y_i|\mu_{1i}) + (1-\pi) f_{NB}(y_i|\mu_{2i}, \alpha). \tag{3}$$

Latent class regression coefficients can be specified for parameters π, μ_{1i}, μ_{2i} and α as

$$\begin{aligned} log(\mu_{1i}) &= \mathbf{z}_i'\boldsymbol{\gamma} \\ log(\mu_{2i}) &= \mathbf{x}_i'\boldsymbol{\zeta} \\ \pi &= \pi \\ log(\alpha) &= -\tau, \end{aligned} \tag{4}$$

where $\boldsymbol{\zeta}$ is a vector of parameters and all the other parameters and variables are as described for the MPois-Pois and MNB-Pois models in equations 7 and 8 of the main article. In line

with standard mixture models, the logit link is not used to model π in equation (4); once π is estimated, a starting value for ρ in the marginal mean model can be obtained by setting $\rho = \text{logit}(\pi)$.

As a complete data likelihood function is needed to implement EM algorithm, we define an indicator variable U_i corresponding to the ith subject as

$$U_i = \begin{cases} 1, & \text{if subject } i \text{ belongs to subpopulation 1} \\ 0, & \text{if subject } i \text{ belongs to subpopulation 2} \end{cases}$$

Thus, U_i has a Bernoulli distribution with parameter π.

$$Pr(U_i = u_i|\pi) = \pi^{u_i}(1-\pi)^{1-u_i}, u_i = 0, 1.$$

The random variable (Y_i, U_i) contains an observed outcome Y_i and a missing variable U_i, and the contribution of (Y_i, U_i) to the complete data likelihood is given by,

$$\begin{aligned} L_{ic}(\pi, \boldsymbol{\gamma}, \boldsymbol{\zeta}, \tau|u_i, y_i, \mathbf{x}_i, \mathbf{z}_i) &= Pr(Y_i = y_i|\boldsymbol{\gamma}, \boldsymbol{\zeta}, \tau, \mathbf{x}_i, \mathbf{z}_i; U_i = u_i)Pr(U_i = u_i|\pi) \qquad (5) \\ &= \Big[\pi f_P(y_i|\boldsymbol{\gamma}, \mathbf{z}_i)\Big]^{u_i}\Big[(1-\pi)f_{NB}(y_i|\boldsymbol{\zeta}, \tau, \mathbf{x}_i)\Big]^{1-u_i} \end{aligned}$$

The likelihood function L_c from n independent counts is the product of each likelihood in equation (5). That is,

$$L_c(\pi, \boldsymbol{\gamma}, \boldsymbol{\zeta}, \tau|\mathbf{u}, \mathbf{y}, \mathbf{x}, \mathbf{z}) = \prod_{i=0}^{n}\Big[\pi f_P(y_i|\boldsymbol{\gamma}, \mathbf{z}_i)\Big]^{u_i}\Big[(1-\pi)f_{NB}(y_i|\boldsymbol{\zeta}, \tau, \mathbf{x}_i)\Big]^{1-u_i}.$$

The corresponding log-likelihood is given by

$$\begin{aligned} \ell_c(\pi, \boldsymbol{\gamma}, \boldsymbol{\zeta}, \tau|\mathbf{u}, \mathbf{y}, \mathbf{x}, \mathbf{z}) &= \sum_{i=0}^{n}[u_i\, logit(\pi) + log(1-\pi)] + \sum_{i=0}^{n} u_i\, log(f_P(y_i|\boldsymbol{\gamma}, \mathbf{z}_i)) \\ &+ \sum_{i=0}^{n}[(1-u_i)log(f_{NB}(y_i|\boldsymbol{\zeta}, \tau, \mathbf{x}_i))] \end{aligned}$$

Given initial parameter values $\boldsymbol{\theta}^{(0)} = (\pi^{(0)}, \boldsymbol{\gamma}^{(0)}, \boldsymbol{\zeta}^{(0)}, \tau^{(0)})$, the E step of EM computes the expected value of ℓ_c conditional on the observed variables and $\boldsymbol{\theta}^{(0)}$.

$$\begin{aligned} E(\ell_c(\pi, \boldsymbol{\gamma}, \boldsymbol{\zeta}, \tau|\mathbf{u}, \mathbf{y}, \mathbf{x}, \mathbf{z})|\boldsymbol{\theta}^{(0)}, \mathbf{y}, \mathbf{x}, \mathbf{z})) &= \sum_{i=0}^{n}[E(u_i|\boldsymbol{\theta}^{(0)}, y_i, \mathbf{x}_i, \mathbf{z}_i)\, logit(\pi) + log(1-\pi)] \\ &+ \sum_{i=0}^{n} E(u_i|\boldsymbol{\theta}^{(0)}, y_i, \mathbf{x}_i, \mathbf{z}_i)\, log(f_P(y_i|\boldsymbol{\gamma}, \mathbf{z}_i)) \\ &+ \sum_{i=0}^{n}[log(f_{NB}(y_i|\boldsymbol{\zeta}, \tau, \mathbf{x}_i))(1 - E(u_i|\boldsymbol{\theta}^{(0)}, y_i, \mathbf{x}_i, \mathbf{z}_i))] \end{aligned}$$

It can be shown that

$$\begin{aligned}E(u_i|\boldsymbol{\theta}^{(0)}, y_i, \mathbf{x}, \mathbf{z}) &= \frac{\pi^{(0)} f_P(y_i|\boldsymbol{\gamma}, \mathbf{z}_i)}{\pi^{(0)} f_P(y_i|\boldsymbol{\gamma}, \mathbf{z}_i) + (1-\pi^{(0)}) f_{NB}(y_i|\boldsymbol{\zeta}, \tau, \mathbf{x}_i)} \\ &\equiv P_i^{(0)}\end{aligned}$$

Thus, the M step maximizes,

$$\begin{aligned}E(\ell_c(\pi, \boldsymbol{\beta}, \boldsymbol{\zeta}, \tau|\mathbf{u}, \mathbf{y}, \mathbf{x}, \mathbf{z})|\boldsymbol{\theta}^{(0)}, \mathbf{y}, \mathbf{x}, \mathbf{z})) &= \sum_{i=0}^{n} [P_i^{(0)}\, logit(\pi) \\ &+ log(1-\pi)] + \sum_{i=0}^{n} P_i^{(0)}\, log(f_P(y_i|\boldsymbol{\gamma}, \mathbf{z}_i)) \\ &+ \sum_{i=0}^{n} [log(f_{NB}(y_i|\boldsymbol{\zeta}, \tau, \mathbf{x}_i))(1-P_i^{(0)})] \\ &= \ell_\pi + \ell_{\boldsymbol{\gamma}} + \ell_{(\boldsymbol{\zeta},\tau)}\end{aligned} \tag{6}$$

To obtain the next estimates in the M step, the three components ℓ_π, $\ell_{\boldsymbol{\gamma}}$ and $\ell_{(\boldsymbol{\zeta},\tau)}$ of the expected log-likelihood in equation (6), can be optimized separately. Maximizing ℓ_π with respect to π gives

$$\pi^{(1)} = \sum_{i=0}^{n} \frac{P_i^{(0)}}{n}.$$

The remaining two components of the expected log-likelihood (i.e., $\ell_{\boldsymbol{\gamma}}$ and $\ell_{(\boldsymbol{\zeta},\tau)}$) correspond to weighted log-likelihoods of generalized linear models and estimation can be performed separately to obtain the next set of parameters $\boldsymbol{\gamma}^{(1)}$, $\boldsymbol{\zeta}^{(1)}$ and $\tau^{(1)}$. Utilizing the parameters ($\pi^{(1)}$,$\boldsymbol{\beta}^{(1)}$,$\boldsymbol{\zeta}^{(1)}$, $\tau^{(1)}$) estimated in the first step, EM again computes and optimizes the expected log-likelihood and continues iterations between the two steps until convergence. The NB-Poisson mixture model estimates of π,$\boldsymbol{\gamma}$ and τ at convergence are then employed as starting values for parameters $\rho = \text{logit}(\pi)$, $\boldsymbol{\xi}$ and τ respectively, in the MNB-Pois model.

3.2 Finding Starting Values using SAS software

If the analyst is using SAS software, PROCs Genmod and FMM may be used to obtain starting values in use of Proc NLMIXED for fitting MPois-Pois and MNB-Pois models. SAS Proc FMM fits standard finite mixture models whereas both Proc FMM and Proc Genmod can be used to fit traditional ZIP and ZINB models. The following table summarizes the source of starting values that we suggest for fitting marginalized count models. These are not the only choices and some trial and error may be necessary. For example, the previous section uses the standard NB-Poisson mixture model to obtain starting values for $\rho = \text{logit}(\pi)$, $\boldsymbol{\xi}$ and τ.

For MPois-Pois and MNB-Pois models, recall that equation (7)

$$\mu_{2i} = \frac{\nu_i - \pi\mu_{1i}}{1-\pi}. \tag{7}$$

Model Source of Starting Parameter Values

Model	$\boldsymbol{\beta}$	$\boldsymbol{\xi}$ and ρ	$\boldsymbol{\gamma}$	τ
MZIP	Neg. Bin.	na	ZIP	na
MZINB	Neg. Bin.	na	ZINB	ZINB
MPois-Pois	MZINB	Pois-Pois	na	na
MNB-Pois	MPois-Pois	NB-Pois	na	NB-Pois

$\rho = logit(\pi)$ is the mixing parameter; na= not applicable

is substituted into the standard finite mixture likelihood. Computational errors resulting in non-convergence may result if the computed μ_{2i} is negative. To lessen the chances of this occurrence, we suggest use of starting values of π less than 0.5, which will be the case for one of the model parts from the standard Pois-Pois model. Additionally, the starting values for $\boldsymbol{\xi}$ are chosen from the corresponding model part from Pois-Pois.

References

Leisch F. FlexMix: A General Framework for Finite Mixture Models and Latent Class Regression in R. J. Statist. Soft. **11** 8 (2004).

Ramaswamy, V, Anderson, A.W, DeSarbo, W.S. A Disaggregate Negative Binomial Regression Procedure for Count Data Analysis. Manage. Sci. **40**(3), 405-417 (1994).

Appendix. SAS Code

```
/* file: Lanark-Allmodels.sas
   paper: Marginalized Mixture Models for Count Data from Multiple Source Populations
   authors: Habtamu K. Benecha, Brian Neelon, Kimon Divaris and John S. Preisser
   what: analysis of caries clinical trial for data described in Stephen et al. 1994
         Fit marginalized mean models reported in Table 7 of article */

options ps=54 ls=80;
libname dat 'c:';
data all;
   set dat.LanarkTwoYear;
   if dmfsinc2b = . then delete /* DMFS increment at two years */;
run;
```

```
* Poisson regression;
proc genmod data=all;
   title 'Poisson Regression: two year DMFS increment';
   model dmfsinc2b = bc calc naf naftmp/ d=poisson;
run;

* Neg. Bin. Regression;
proc genmod data=all;
   title 'Negative Binomial Regression: two year DMFS increment';
   model dmfsinc2b = bc calc naf naftmp/ link=log dist=negbin;
run;

* Traditional ZIP model;
proc genmod;
title "traditional ZIP model from GENMOD";
   model dmfsinc2b = bc calc naf naftmp/ link=log dist=ZIP;
   zeromodel bc calc naf naftmp;
run;

* Traditional ZINB model;
proc genmod;
title "traditional ZINB model from GENMOD";
   model dmfsinc2b = bc calc naf naftmp/ link=log dist=ZINB;
   zeromodel         bc calc naf naftmp;
run;

* marginalized ZIP model;
title "marginalized ZIP model from NLMIXED";
proc nlmixed data= all gconv=1E-12;
parms a0=-1.15 a1=-1.26 a2=0.06  a3=0.05 a4=-0.03 /* use ZIP infl model estimates */
      b0=1.22 b1=0.76 b2=-0.20 b3=-0.07 b4=-0.06; /* use Poisson regr estimates */
linpinfl = a0 + a1*bc + a2*calc + a3*naf + a4*naftmp;
infprob = 1/(1+exp(-linpinfl)); /* inflation probability for excess zeros */
nu = exp(b0 + b1*bc + b2*calc + b3*naf + b4*naftmp); /* nu is the marginal mean */
lambda = nu/(1-infprob); /* transform from marg. mean to susceptible class mean */
if dmfsinc2b=0 then prob = infprob + (1-infprob)*exp(-lambda);
if dmfsinc2b=0 then loglike = log(prob);
else loglike = log((1-infprob)) + dmfsinc2b*log(lambda) - lambda - lgamma(dmfsinc2b+1);
model dmfsinc2b ~ general(loglike);
run;
```

```
* marginalized ZiNB;
title "marginalized ZINB model from NLMIXED";
proc nlmixed data= all gconv=1E-12;
parms a0=-2.07 a1=-2.45 a2=-0.24 a3=0.24 a4=0.21 phi=0.63 /* use ZINB infl est */
      b0= 1.20 b1= 0.76 b2=-0.19 b3=-0.06 b4=-0.03; /* use NB regr estimates */
linpinfl = a0 + a1*bc + a2*calc + a3*naf + a4*naftmp;
psi = 1/(1+exp(-linpinfl));
nu = exp(b0 + b1*bc + b2*calc + b3*naf + b4*naftmp);
mu = nu/(1-psi);
alpha = 1/phi;
theta = 1/(1+(mu/alpha));
if dmfsinc2b=0 then loglike =log(psi + (1-psi)*(theta**alpha));
else loglike = log(1-psi) + lgamma(dmfsinc2b+alpha) - lgamma(alpha)
      + dmfsinc2b*log(1-theta)+alpha*log(theta) - lgamma(dmfsinc2b+1);
model dmfsinc2b ~ general(loglike);
run;

***  Standard Pois-Pois regression model fitted using Proc FMM  *******;
proc fmm data=all /* fitdetails itdetails */ gconv=1E-10;
   title "Standard Poisson-Poisson mixture model";
   model dmfsinc2b = bc calc naf naftmp/dist=Poisson;
   model    + bc calc naf naftmp/dist=Poisson;
run;

*********************************************************;
**********  MPois-Pois                            ******;
*********************************************************;
**** Use MZINB betas as starting vals for the b's   *****;
**** and Standard Pois-Pois latent vals for the z's *****;
*********************************************************;
* use pi=0.32, or rho = logit(0.32)=-0.75;
* use gconv option to avoid gradient greater than 1e-3;
proc nlmixed data=all gconv=1E-12;
title 'Lanarkshire Data: marginalized Poisson-Poisson regression model';
title2 'Start vals are MZINB for betas and Pois-Pois for zetas';
parms b0=1.21 b1=0.76 b2=-0.19 b3=-0.06 b4=-0.03
      z0=2.13 z1=0.51 z2=-0.24 z3=-0.15 z4=-0.08 rho=-0.75;
nu =exp(b0 + b1*bc + b2*calc + b3*naf + b4*naftmp);
mu1=exp(z0 + z1*bc + z2*calc + z3*naf + z4*naftmp);
pi = 1.0/(1 + exp(-rho));
mu2 = (nu - pi*mu1)/(1-pi);
part1 = pi*exp(-mu1)*(mu1**dmfsinc2b);
```

```
part2 = (1-pi)*exp(-mu2)*(mu2**dmfsinc2b);
like = (part1 + part2)/gamma(dmfsinc2b+1);
loglik = log(like);
model dmfsinc2b ~ general(loglik);
estimate 'pi mixing' pi;
run;

***  Standard NB-Pois regression model fitted using Proc FMM   ****;
proc fmm data=all gconv=1E-12;
   title "Standard NB-Poisson mixture model";
   model dmfsinc2b = bc calc naf naftmp /dist=negbin;
   model    + bc calc naf naftmp/dist=Poisson;
run;
*********************************************************************************;
****    Marginalized NB-Poisson mixture model                                ***;
*********************************************************************************;
* use Mpois-Pois starting vals for betas, NB-Pois vals for latent model and tau  ***;
*********************************************************************************;
proc nlmixed data=all gconv=1E-12;
title "Lanarkshire Data: Marginalized NB-Poisson regression model";
title2 'Mpois-Pois starting vals for betas';
title3 "NB-Pois starting vals for zetas, rho (mixing parm) and tau (dispersion)";
parms b0=1.30 b1=0.66 b2=-0.26 b3=-0.14 b4=-0.06   /* tau=log(phi), phi=0.71 */
      z0=-1.31 z1=3.5 z2=-0.09 z3=-0.10 z4=-0.44  rho=-1.85 tau =-0.33;
nu =exp(b0 + b1*bc + b2*calc + b3*naf + b4*naftmp); /* marginal mean */
mu1=exp(z0 + z1*bc + z2*calc + z3*naf + z4*naftmp); /* Poi */
pi = 1/(1 + exp(-rho));
mu2 = (nu - pi*mu1)/(1-pi); /* NB mean not being modeled */
alpha = exp(-tau);
theta = 1/(1+(mu2/alpha));
lnpart1 = log(pi)  -mu1 + dmfsinc2b*log(mu1); /* Poisson part */
lnpart2 = log(1-pi) + lgamma(dmfsinc2b+alpha) - lgamma(alpha) +
             dmfsinc2b*log(1-theta) + alpha*log(theta); /* NB part */
like = (exp(lnpart1) + exp(lnpart2))/gamma(dmfsinc2b+1);
loglike = log(like);
model dmfsinc2b ~ general(loglike);
estimate 'mixing probability' pi;
estimate 'scale parameter, phi' 1/alpha;
run;
```

Table 1: Percentages of converged marginalized models fitted to data generated from the MZIP model with 10,000 replications.

Sample Size	MPois-Pois	MNB-Pois	MZIP	MZINB
100	38.6	17.6	99.8	88.2
200	38.9	17.4	99.8	90.0
500	42.0	22.6	99.8	86.8
1000	46.4	23.9	99.8	88.6

Table 2: Percent relative median biases of estimates of β_1, β_2 and β_3 from marginalized mixture models fitted to data generated from the MZIP model with 10,000 replications.

Sample Size	Parameter	MPois-Pois	MNB-Pois	MZIP	MZINB
	β_1	15.36	-34.29	0.05	0.08
100	β_2	-18.25	7.39	0.18	0.05
	β_3	-20.26	3.58	0.13	0.08
	β_1	-15.30	-19.20	-0.08	-0.10
200	β_2	-21.40	0.02	0.04	0.0
	β_3	-21.34	-1.24	0.56	0.44
	β_1	17.61	-10.25	0.17	0.25
500	β_2	-22.93	-4.39	-0.04	-0.08
	β_3	-21.55	-4.73	0.02	0.06
	β_1	18.12	-4.53	0.07	0.09
1000	β_2	-23.13	-4.55	0.14	0.18
	β_3	-22.72	-6.30	0.02	0.01

Table 3: Type I error for the estimate of β_1 from marginalized models fitted to data generated from the MZIP model with 10,000 replications.

Sample Size	MPois-Pois	MNB-Pois	MZIP	MZINB
100	0.192	0.396	0.054	0.052
200	0.316	0.318	0.051	0.049
500	0.710	0.184	0.050	0.049
1000	0.948	0.199	0.051	0.050

Table 4: Coverages of 95% confidence intervals of estimates of β_1, β_2 and β_3 from marginalized models fitted to data generated from the MZIP model with 10,000 replications.

Sample Size	Parameter	MPois-Pois	MNB-Pois	MZIP	MZINB
	β_1	88.3	64.6	94.6	94.9
100	β_2	73.6	70.3	94.4	94.6
	β_3	81.6	76.9	95.1	95.3
	β_1	84.3	68.5	95.0	95.2
200	β_2	54.1	74.7	95.0	95.2
	β_3	67.9	78.9	95.0	95.2
	β_1	80.9	72.4	95.1	95.2
500	β_2	18.7	73.8	95.1	95.2
	β_3	37.1	76.6	95.3	95.4
	β_1	89.3	77.3	94.9	95.1
1000	β_2	3.5	69.0	95.1	95.2
	β_3	12.2	76.5	95.0	95.1

Table 5: Percentages of converged marginalized models fitted to data generated from the MZINB model with 10,000 replications.

Sample Size	MPois-Pois	MNB-Pois	MZIP	MZINB
100	84.8	79.7	99.7	96.5
200	84.0	90.1	99.7	98.7
500	87.9	94.8	99.7	99.6
1000	97.0	96.0	99.7	99.7

Table 6: Percent relative median biases of estimates of β_1, β_2 and β_3 from marginalized mixture models fitted to data generated from the MZINB model with 10,000 replications.

Sample Size	Parameter	MPois-Pois	MNB-Pois	MZIP	MZINB
100	β_1	-9.54	-1.42	-2.88	-3.08
	β_2	0.44	9.13	7.85	-0.59
	β_3	3.03	-0.08	3.68	-0.56
200	β_1	-4.49	-0.38	-1.61	-0.50
	β_2	-4.85	4.33	4.82	-0.75
	β_3	0.22	-0.04	2.22	-0.76
500	β_1	-3.51	-0.33	-0.28	-0.36
	β_2	-7.22	1.18	2.90	-0.37
	β_3	-2.67	0.16	1.10	-0.17
1000	β_1	-2.87	-0.15	0.28	-0.13
	β_2	-7.78	0.38	3.01	0.07
	β_3	-2.05	0.02	1.50	0.15

Table 7: Type I error for the estimate of β_1 from marginalized models fitted to data generated from the MZINB model with 10,000 replications.

Sample Size	MPois-Pois	MNB-Pois	MZIP	MZINB
100	0.397	0.116	0.312	0.064
200	0.380	0.074	0.313	0.054
500	0.349	0.056	0.313	0.052
1000	0.345	0.053	0.326	0.050

Table 8: Coverages of 95% confidence intervals of estimates of β_1, β_2 and β_3 from marginalized models fitted to data generated from the MZINB model with 10,000 replications.

Sample Size	Parameter	MPois-Pois	MNB-Pois	MZIP	MZINB
	β_1	53.4	89.0	58.1	93.6
100	β_2	60.7	84.1	70.9	92.8
	β_3	62.5	91.7	66.0	93.9
	β_1	54.4	92.5	57.6	94.4
200	β_2	61.9	85.2	69.4	93.5
	β_3	61.6	94.0	65.5	94.7
	β_1	54.1	94.0	56.9	94.7
500	β_2	60.6	85.3	69.1	95.0
	β_3	61.2	95.1	63.8	95.1
	β_1	56.3	94.6	55.9	95.1
1000	β_2	57.0	83.6	68.4	94.9
	β_3	62.3	95.1	64.7	94.9
